# Impact of video instructions and additional hands-on instructions on the dental flossing performance – an observational study

**DOI:** 10.1007/s00784-024-06070-x

**Published:** 2024-11-28

**Authors:** Katja Jung, Sophie-Charlotte Schmidt, Benedikt Luka, Nadine Schlueter, Carolina Ganss

**Affiliations:** 1 Department of Restorative Dentistry and Endodontology, Dental Clinic, Justus-Liebig-University of Giessen, Giessen, Germany; 2 Present Address: Department of Operative Dentistry, Endodontics, and Paediatric Dentistry, Section of Cariology, Medical Centre of Dentistry, Phillips-University Marburg, Marburg, Germany; 3 Hannover Medical School, Department of Conservative Dentistry, Periodontology and Preventive Dentistry, Hannover, Germany

**Keywords:** Dental flossing, Flossing technique, Preventive dentistry, Video-observation, Video instructions, Instruction technique

## Abstract

**Objectives:**

This study aimed to evaluate the effectiveness of an instruction-video, with and without additional hands-on instruction, in teaching young adults proper flossing performance.

**Materials and Methods:**

This randomised intervention and observational study included 94 participants, 24 (23; 25) years, receiving an instruction-video with (group 1) or without additional hands-on instruction (group 2). The flossing performance was assessed by videotaping before instruction (T1), after instruction and one week of practice (T2), as well as after two weeks of practice (T3). Parameters of interest were number of interdental spaces reached, adaptation to mesial and distal surfaces, flossing technique, systematic approach and handling combined with self-assessment of flossing skills and time spent, assessed by questionnaires.

**Results:**

Additional hands-on instruction did not lead to a significant improvement in any parameter compared to video-instruction alone. At T1, the participants reached many interdental spaces, but only two managed to floss their entire dentition correctly. After video instruction (T2), the participants reached more interdental spaces (p = 0.039) using a systematic approach and improved adaptation, technique and handling (*p < *0.001 each). Parameters improved again at T3, resulting in 42 participants flossing their entire dentition correctly. After instruction participants found flossing less difficult, although they estimated it took more time.

**Conclusion:**

Video-instruction significantly improved flossing performance, whereas hands-on instruction had no additional benefit. This indicates that video-instruction could be an effective and timesaving tool to improve flossing performance.

**Clinical relevance:**

The results emphasize the importance of appropriate teaching-methods in dental prophylaxis and highlight the potential of video-instructions to improve interdental cleaning skills significantly.

**Supplementary Information:**

The online version contains supplementary material available at 10.1007/s00784-024-06070-x.

## Introduction

Effective oral hygiene is a cornerstone in promoting oral health. In addition to removing plaque with toothbrushes this also includes interdental hygiene, for which aids such as interdental brushes and dental floss can be used [1].

Despite the widespread recommendation, actual usage of dental floss is generally low and varies between the studied countries and populations [2–5]. The reasons for the low implementation have not yet been sufficiently investigated but are likely to be complex. On the one hand, psychological constellations such as a lack of self-efficacy and planning may be relevant [6] and on the other hand, factors such as laziness, lack of guidance and confidence in one's own abilities as well as problems concerning manual dexterity [7] may influence the regular use of dental floss.

Additionally there is also some controversy about the general benefits of flossing. Some studies have found dental floss to be effective in cleaning approximal surfaces [8, 9]. Other studies have associated the additional use of dental floss with a reduction in the total amount of plaque [10, 11] and, in the long term, with the prevention of root caries [12] and tooth loss [13]. In contrast, systematic reviews found only little evidence for a reduction in gingivitis [14–16], and only weak and very unreliable evidence that flossing in combination with tooth brushing was associated with an additional reduction in plaque [14–16] or caries [15, 17].

Due to a lack of evidence, the benefits of flossing have been questioned and the scientific conclusions and recommendations range from “flossing is an effective adjunct to toothbrushing, as the important benefits outweigh any potential harms” [15] to “a routine recommendation to use floss is not supported by scientific evidence“ [18]. However, a critical analysis of the existing literature shows, that many studies on the effectiveness of dental floss have significant methodological flaws and their conclusions are unreliable [9, 15, 19].

The main criticisms are small numbers of participants [17] and short study durations [16]. In addition, many studies were conducted with rather low baseline levels of gingival inflammation, which weakens the benefit of dental floss [9, 16]. Another point is, that many studies [14–16] use plaque indices such as the Turesky modified Quigley and Hein Index (T-QHI) [20], the Silness and Loe Plaque-Index [21] or the Rustogi modified Navy-Plaque-Index (RMNPI) [22]. These indices assess the entire smooth surface of a tooth and therefore have limited validity for separate plaque quantification of areas close to the interdental space.

Furthermore, almost none of the studies included in the reviews described flossing instructions or the extent to which participants were able to floss correctly. The finding that regular professional flossing led to an effective reduction in proximal caries for children with low fluoride exposures, while self-use showed no effect [17], suggests that these methodological weaknesses may mask existing effects of interdental hygiene.

In light of a study which showed that over 90% of the participants were unable to use dental floss correctly [23], it is reasonable to conclude that the limited effects of flossing may be merely due to poor implementation rather than actual uselessness.

It is often assumed, that flossing is difficult to learn, although only little has been published on the topic. However, two older studies have shown that an individual as well as a group instruction which included a 20-min video lecture followed by a 25-min practice-session parallel to the video, led to an improvement in flossing skills of over 80% [24]. The combination of both individual and group instruction methods even led to an improvement of over 90% [25]. These results show that correct flossing can be taught to laypersons, although time and human effort required in these studies were high. Summing up, still very little is known about how a correct flossing technique can be achieved.

The aim of this study was therefore to investigate whether a short video instruction can improve flossing skills and if an additional hands-on instruction has an incremental benefit. The criteria used were the number of correctly flossed interdental spaces and the correct implementation of the flossing technique as well as the improvement in self-assessment.

## Participants, materials and methods

The study was a randomized intervention and observational study with healthy volunteers. It was conducted according to the principles of the Declaration of Helsinki and the principles of Good Clinical Practice [26]. Prior to commencement the study was approved by the local ethics committee (ethics committee of the University of Giessen, ref. no. 156/15). A written informed consent was obtained from all participants.

The study was conducted on students and former university students who were not affiliated with medicine or dentistry. For recruitment purposes, information sheets were placed in central locations around the university.

Inclusion criteria were: willingness to participate, written informed consent, age of 18 years or older.

Exclusion criteria were: absence of more than four permanent teeth (except wisdom teeth), bridges, gap dentition, tooth mobility > 1 (Grace & Smales Mobility Index [27]), multiple recessions with an extension of more than one third of the root length, fixed orthodontic appliances (including retainers), mental and/or physical disability which limited the ability to perform interdental hygiene.

### Study Procedure

At the first session, participants were informed about the content of the study and received an information sheet as well as the informed consent form. The inclusion and exclusion criteria were reviewed and, if present, calculus was removed. Eligible subjects were included in the study after they had given their informed consent.

After enrolment, participants were allocated to two intervention groups by using a randomisation list (Excel 2013, Version 15.0).

At baseline (T1), flossing performance was filmed (JVC, GR-D230E, Friedberg, Hessen, Germany) through a one-way mirror in.avi format (DVCPRO) at 25 frames per second. Participants were left alone in the room during all video recordings. An identical dental floss dispenser (Johnson & Johnson® Reach Waxed Dentotape, New Brunswick, NJ, USA), a sink, soap and paper towels were available at each appointment. In a standardised dialogue, participants were asked to floss habitually, i.e. not more carefully than usual. Those who had never flossed before were asked to do so at their discretion. Afterwards, the participants completed a questionnaire.

After baseline filming, the participants watched an instruction video lasting around 4 min, which had been created especially for this study. In close-up it illustrates the correct application of dental floss into the interdental space (sliding over the proximal contact, applying the floss to one of the two tooth surfaces, several vertical movements, applying to the other tooth surface, vertical movements) and the correct handling (winding technique). Additionally, the film demonstrates how flossing should be done in a systematic way (from 17 to 47). The video was partly subtitled and accompanied by spoken text.

Group 1 received additional hands-on instruction in front of a mirror after watching the instructional video. They received detailed demonstration about the correct way to wrap the floss around the fingers and the correct hand position. Participants were then asked to self-perform flossing while being re-instructed until the flossing technique seemed accurate. Group 2, on the other hand, received no hands-on instruction.

At the end of the session, all participants were asked to practice what they had learned at home for one week.

At the second appointment (T2, one week after T1), all participants were filmed again and completed the same questionnaire again as at T1.

The third appointment (T3, one week of practice after T2) followed the pattern of session 2. At the end of the study, participants in group 2 were offered a hands-on instruction on demand.

**Fig. 1:**
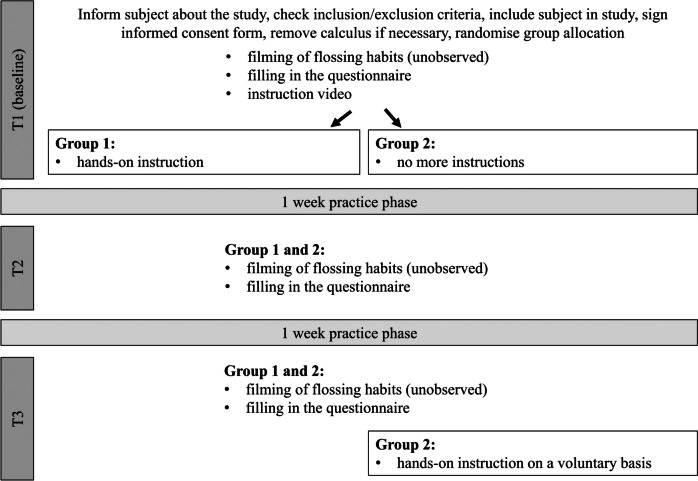
Flow chart of the study procedure

The study procedure is shown in Fig. 1.

### Questionnaires

At the first visit, general information was collected on gender, age, frequency of use of oral hygiene aids and whether flossing technique had ever been taught. At all three visits (T1-T3) ten-point scales (strongly disagree—strongly agree) with the following items were provided: flossing is easy to use, flossing is time consuming, I reach all interdental spaces, I reach all anterior spaces, I reach all posterior spaces.

### Video analysis

After all participants had completed their three appointments the video analysis was done with a video analysis software (Interact Version 9.6.1.170, Mangold International, Arnstorf, Germany) based on the Flossing Dexterity Index [28, 29] and as described in detail in [30]. A count coding system was developed to measure continuous temporal events; all videos were coded exhaustively, i.e., all seconds of the observation session were coded by using a continuous temporal behavioural sampling [31, 32]. Twenty-six interdental spaces were assessed. Four codings were defined for the flossing technique:

#### Movements

Flossing movements were coded as “vertical” or “horizontal”, if the participant performed this movement in the interdental space two or more times. A single movement was coded as “in–out”. “Pull through” was coded if the floss was not removed again over the contact point, but pulled out to the side. The simultaneous insertion of floss into multiple proximal contacts and the associated wrapping of the teeth was referred to as “looping”. Movements which did not correspond to any of these categories were coded as “unspecific”.

#### Reached interdental spaces

An interdental space was coded as “reached” as soon as the floss had passed the contact point.

#### Adaptation

The correct adaptation of floss to the mesial and distal surfaces of an interdental space was coded if the floss was adapted first to one and then to the other tooth surface regardless of the sequence. The adaptation was coded as “correct”, if the adaptation was performed correctly in all interdental spaces.

#### Handling of the floss

Handling was coded as correct if the floss was unwound and rewound onto the fingers each time after reaching an interdental space, so that a clean piece of dental floss was used each time and the dental floss was then guided over the contact point with gliding movements. The handling was also coded as “correct” if it was carried out correctly for all interdental spaces.

The flossing performance is classified as correct if at least 24 interdental spaces were reached, the floss was adapted to the mesial and distal tooth surfaces in all interdental spaces reached and both tooth surfaces were cleaned with vertical movements in more than 90%. In addition, the handling of the dental floss must be correct in the entire dentition.

Besides the parameters for technique, a score was assigned for systematics. Systematic flossing was defined when approaching neighbouring interdental spaces consecutively regardless of the starting area. For quantification purposes, one score point was assigned for each consecutively reached interdental space, which added up to a total score of 24, if flossing was perfectly systematic.

### Inter- and intra-examiner agreement and blinding

The analysis was carried out by S-C.S. Before starting the analysis, several training sessions were conducted using videos from a previous study [23]. After the training phase, the consistency of the ratings was assessed by using intrarater reliability. For this purpose, 10 randomly selected videos were analysed at two different time points, resulting in a kappa value ĸ = 0.84/1/0.9/1 for interdental space/handling/technique/adaptation.

Blinding was achieved by analysing the videos in random order and at different time points after completion of clinical data collection.

### Statistical analysis

The sample size calculation (G*Power Version 3.1.9.2) was based on data from [Radentz et al., 1975]. A 25% increase in the number of teeth on which dental floss was correctly used, was regarded as a clinically relevant improvement. With an alpha of 0.05 and a beta of 0.80, this resulted in 40 participants per group. To compensate possible dro*p-*outs it was planned to recruit a total of 100 subjects.

Statistics were performed using IBM SPSS Statistics version 25 (IBM Deutschland GmbH, Ehningen, Germany). The Kolmogorov–Smirnov test revealed a significant deviation of the Gaussian distribution for most of the data. Therefore, non-parametric tests were used (Wilcoxon test, Mann–Whitney-U-test, chi-squared test, Spearman correlation). Values are given as median and 95% confidence intervals obtained by bootstrapping (method of sampling: simple, number of samples: 1000), the level of significance was set at 0.05. Odds ratio were computed for the relationship of correct flossing performance and flossing frequency prior to the study (≥ once a week, < once a week), prior instructions (yes/no) as well as gender (female/male).

The graphical presentation is done with boxplots showing the minimum value, first quartile, median, third quartile, and maximum value. Outliers (circles) are cases lying more than 1.5 box-lengths outside of the box, and extreme values (asterisks) more than 3 box-lengths.

## Results

We included 97 participants, whereas three of them did not attend all the events. This results in a total number of 94 participants (24.3 ± 3.1 years; Group 1: 30 females and 17 males, 24 (23; 25) years old; Group 2: 34 females and 13 males, 24 (23; 24) years old; p for the comparison of gender and age 0.507 and 0.443, respectively). The initial conditions in terms of frequency of dental flossing at home and previous instructions were very comparable for both groups. In group 1, 22 participants reported that they flossed weekly or more often, while 25 did so less often; in group 2, the figures were 21 and 26, respectively (*p* = 1.00). Eleven participants in group 1 and 13 in group 2 stated that they had already received instruction in the correct use of dental floss, whereas 36 and 34 respectively had not yet received any instruction (*p* = 0.813).

### Effects of the instruction methods

With regard to the observed use of dental floss, the hands-on instruction showed no additional benefit compared to the video instruction alone for any of the parameters investigated (the data are shown in detail in supplementary Table 1).

The participants in both groups were able to reach many interdental spaces even before the instruction, but they were unsuccessful at flossing them adequately. The instruction generally resulted in significant improvements in flossing performance (see below). Regardless of whether they had received hands-on instruction, the participants implemented the correct technique after the instruction much better and showed a significantly more systematic approach.

Furthermore, the participants in both groups assessed the various aspects of flossing in the questionnaires similarly at all time points.

This finding suggests that the two groups can be combined for a clearer presentation of the individual results and a more in-depth analysis of influencing factors.

### Questionnaires

**Fig. 2:**
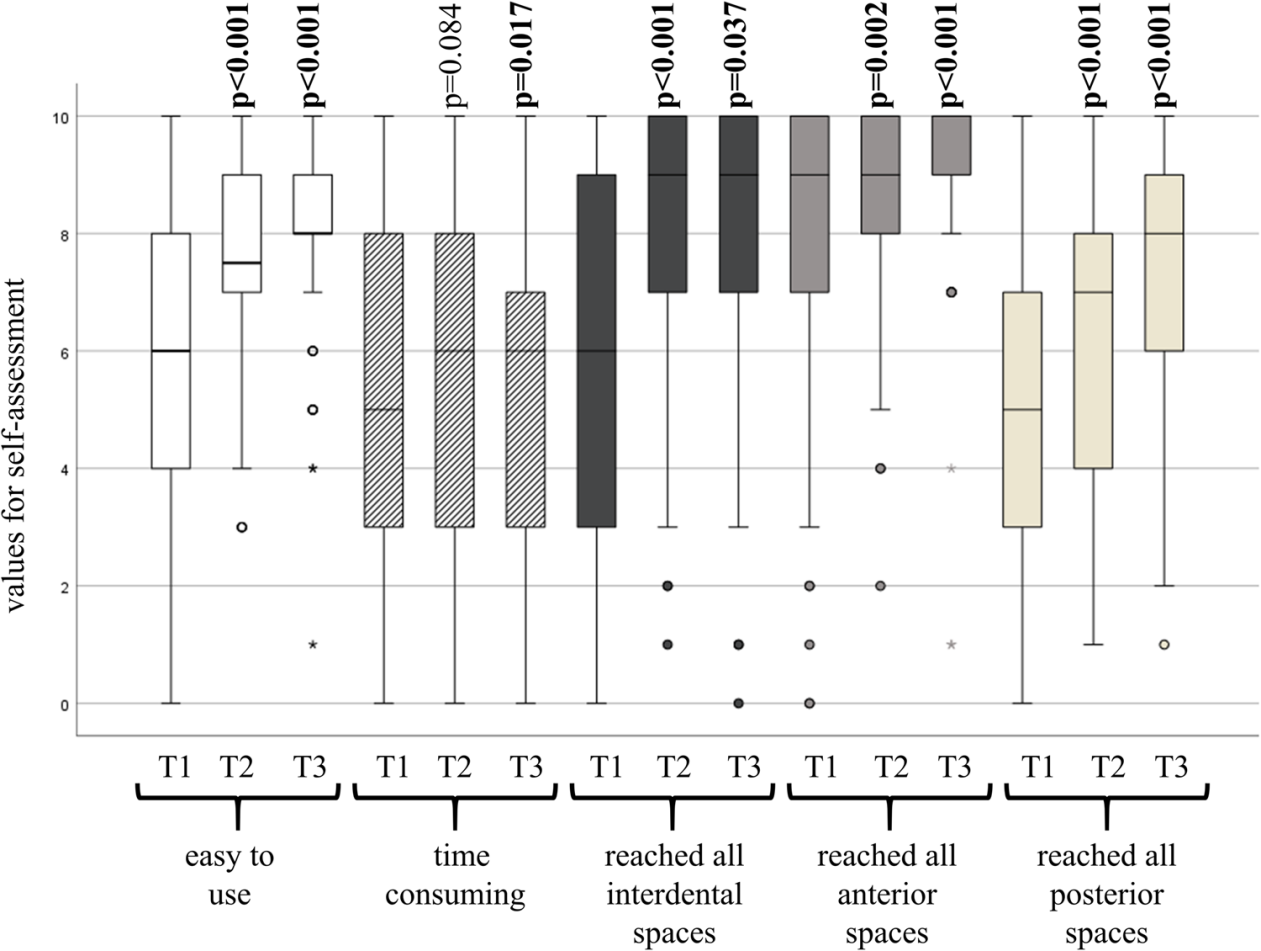
Analogue scale scores at T1, T2 and T3. Significant changes compared to the previous appointment are marked in bold

Self-assessment questionnaire at baseline (T1), showed a wide range of scores in terms of difficulty, time required, and accessibility of areas. After instruction and implementation (T2 and T3) the participants considered the application as less difficult and believed that they could reach more interdental spaces. These effects persisted with prolonged use (T3). In contrast, the perception of the time required did not change significantly; in fact, at T3, participants judged the time required to be longer (Fig. 2).

### Observational data

#### Interdental spaces reached

**Fig. 3:**
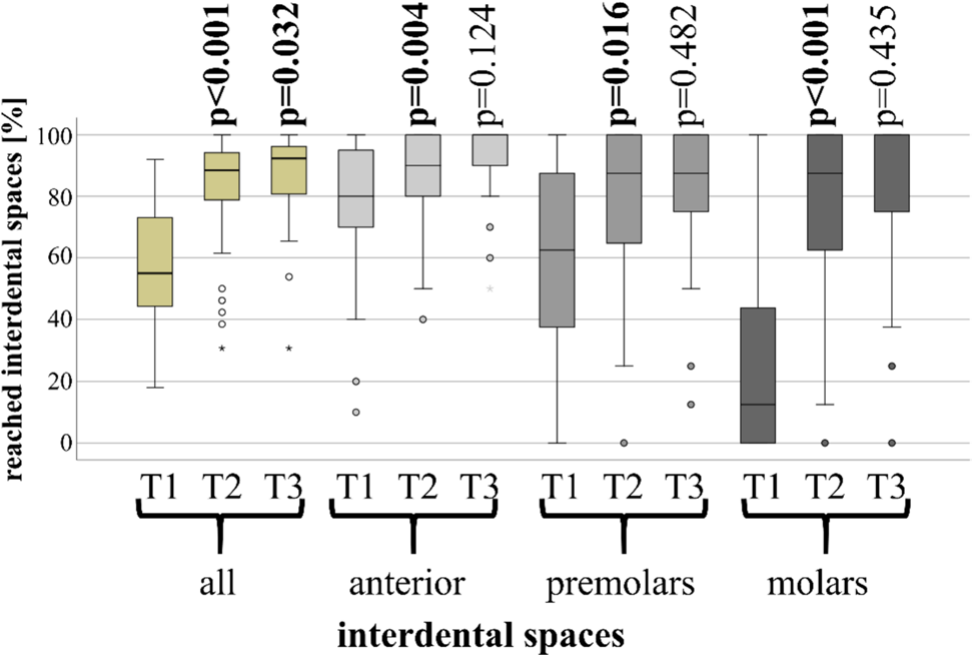
Reached interdental spaces at T1, T2 and T3 for all areas of the dentition as well as for the anterior/canine teeth, premolars and molars separately. Participants who had already reached all interdental spaces at T1 were excluded (included participants: n = 43). Significant changes compared to the previous appointment are highlighted in bold

At baseline (T1) approximately half of the participants (54.3%) were able to reach almost all (24 or more) interdental spaces, while the remainder (45.7%) showed an even distribution over the entire range from 1 to 23 spaces (median 14 interdental spaces). Furthermore, participants showed the highest ability to reach their interdental spaces in the anterior region, but struggled with accessing the interdental spaces in the molar region, as depicted in Fig. 3.

Participants who reached almost all interdental spaces at baseline continued to maintain high levels of reach at the two subsequent observations points, with no significant changes observed. Conversely, those who initially did not reach all interdental spaces at T1 improved significantly from T1 to T2 and again from T2 to T3. This effect was observed in all tooth groups, but especially for the molars. Initially, 40.4% of these participants reached half or even less of their interdental molar spaces and 18.1% were unable to reach any interdental molar spaces at all. After instruction, the proportion of these participants reaching half or less of their molar interdental spaces decreased to 9.7% at T2 and diminished further to 7.5% at T3, with only 1.1% unable to reach any interdental molar spaces at T3.

#### Systematic approach

**Fig. 4:**
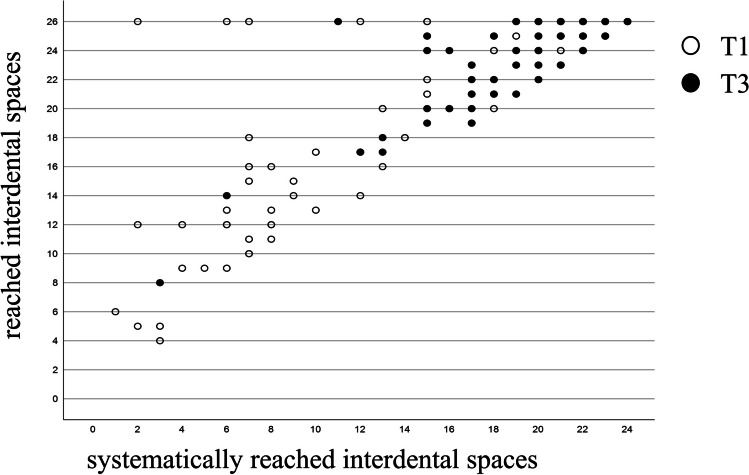
Correlation between systematic scores and reached interdental spaces (unfilled circles T1; black circles T3)

At baseline, a systematic score of 18 (14; 20) was achieved. As a result of the instruction, the participants not only reached more interdental spaces at T3, but also proceeded more systematically overall (systematic score 21 (21; 22)). A significant association was found between the systematic score and the interdental spaces reached (T1: r = 0.755, *p < *0.001; T3: r = 0.691; *p < *0.001; Fig. 4).

#### Flossing technique

**Fig. 5:**
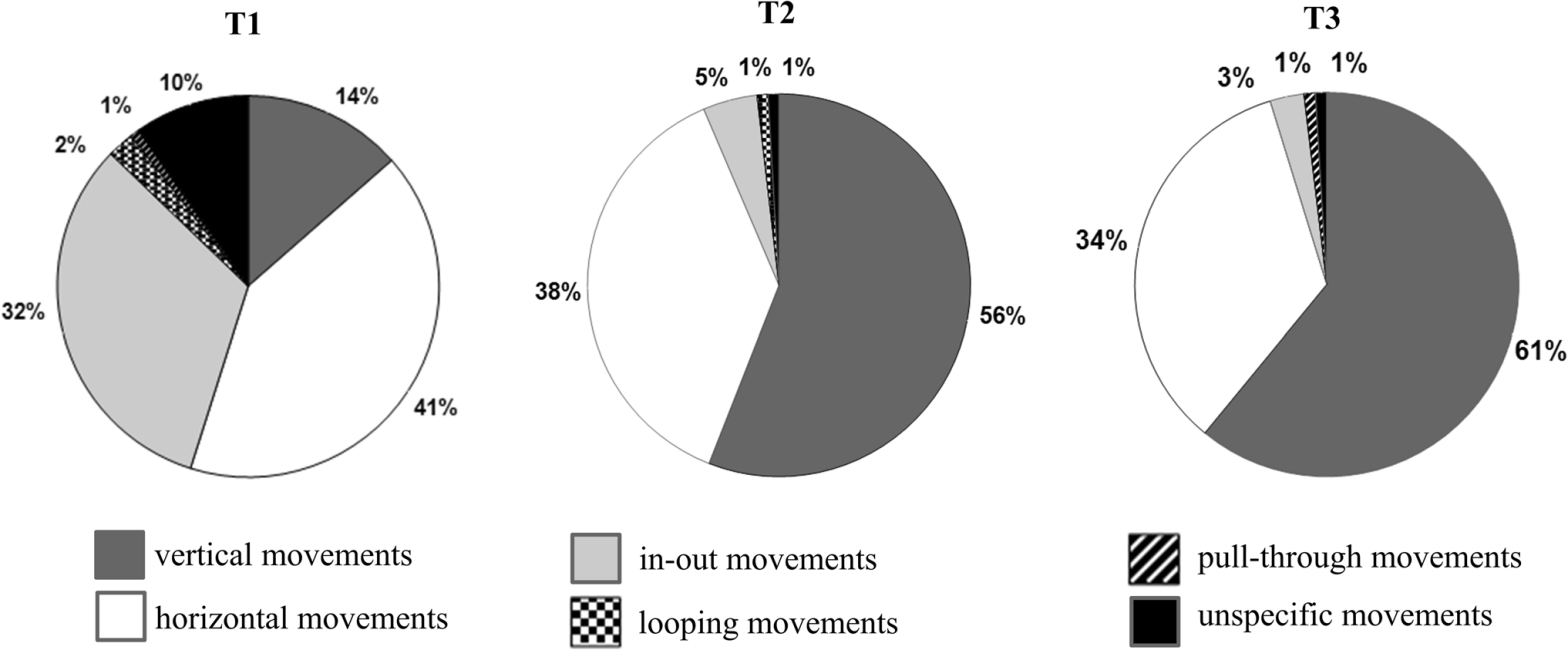
Percentage of different techniques across all participants and interspaces at T1 to T3

At baseline, almost no one was able to perform the correct vertical movements, not even those who initially reached all interdental spaces. Rather horizontal movements, single pull-throughs, single in–out or unspecific movements were used. In some cases, several different movements were implemented in one interdental space, sometimes simultaneously in two interdental spaces. After instruction, mainly repeated horizontal or vertical movements were performed (Fig. 5).

#### Implementation of the correct flossing technique

At the beginning of the study (T1), about half of the participants (54.3%) were able to reach 24 or more interdental spaces. But only ten were able to adapt the floss on the mesial and distal surfaces of these interdental spaces. Out of these ten participants only three applied the vertical flossing technique to 90% or more of the interdental spaces reached. One of them handled the floss incorrectly. Thus, only two participants were able to floss their entire dentition correctly.

After instruction (T2), a significant increase in the number of interdental spaces reached could be seen, in addition to a notable improvement in other parameters such as adaptation of the floss to the mesial and distal tooth surface, flossing technique and handling (*p < *0.001). All parameters significantly improved further at T3, except for the reached interdental spaces, leading to 42 (44.7%) participants flossing their entire dentition correctly (Table 1).

**Table 1:** Number (n) of participants (percentage) who accumulatively fulfilled the listed criteria for a correct flossing performance

Accumulation of criteria for the correct use of dental floss	T1 (n)	T2 (n)	T3 (n)	*p-*values 1 T1 to T2	*p-*values 2 T2 to T3	*p-*values 3 T1 to T3
24 or more interdental spaces reached	**51** (54.3%)	**60** (63.8%)	**65** (69.1%)	***p* = 0.039**	*p* = 0.275	***p* = 0.004**
** + **mesial/distal adaption	**10** (10.6%)	**53** (56.4%)	**62** (66%)	***p < *0.001**	***p* = 0.05**	***p < *0.001**
** + **vertical technique in more than 90% of the interdental spaces reached	**3** (3.2%)	**30** (32.5%)	**43** (45.7%)	***p < *0.001**	***p < *0.002**	***p < *0.001**
** + **correct handling	**2** (2.1%)	**30** (32.5%)	**42** (44.7%)	***p < *0.001**	***p < *0.003**	***p < *0.001**

#### Time

After instruction, the amount of time required was doubled (T1: 100 s (33; 380), T2: 215 s (93; 723), p ≤ 0.001). Further practice did not reduce the required time (T3: 226 s (85; 896), n. s. compared to T2). This effect was particularly evident in those who had not reached all interdental spaces at baseline (T1: 79 s (34; 380), T2: 208 s (93; 452), p ≤ 0.001, T3: 212 s (85; 480), n. s., n = 51).

### Influence parameters

#### Previous flossing and previous instructions

The two participants who used a correct flossing technique at baseline of the study reported flossing daily. Out of all the other participants, who had not yet fully mastered the correct technique, 41 participants reported routinely flossing more than once a week and 51 participants less than once a week. After instruction (T2), especially those who flossed less than once a week were able to implement the correct technique better than those who flossed more than once a week (OR: 5.6; *p* = 0.001). This continued at T3, with a still significant improvement.

In contrast, whether someone had already received instructions before the study had no significant effect on the implementation of the correct technique (Table 2).

**Table 2:** Cross tabulations for the correct flossing performance at T1, T2 and T3; odds ratios (OR) as well as *p-*values

	correct flossing performance			OR (95% CI)	*p-*value
		**flossing frequency prior to the study**			
		≥ once a week (ref.)	< once a week		
T1	yes no	2 41	0 51	6.2 (0.3; 132.8)	*p* = 0.243
T2	yes no	21 22	43 8	5.6 (2.1; 14.7)	***p* = 0.001**
T3	yes no	18 25	34 17	2.8 (1.2; 6.4)	***p* = 0.017**
		**prior instructions**			
		yes (ref.)	no		
T1	yes no	1 23	1 69	3.0 (0.2; 50.0)	*p* = 0.444
T2	yes no	8 16	22 48	1.1 (0.4; 2.9)	*p* = 0.863
T3	yes no	11 13	31 39	1.06 (0.4; 2.7)	*p* = 0.895
		**gender**			
		female (ref.)	male		
T1	yes no	1 63	1 29	0.5 (0.03; 7.6)	*p* = 0.588
T2	yes no	23 41	7 23	1.8 (0.7; 5.0)	*p* = 0.225
T3	yes no	31 33	11 19	1.6 (0.7; 4.0)	*p* = 0.286

#### Gender

**Fig. 6:**
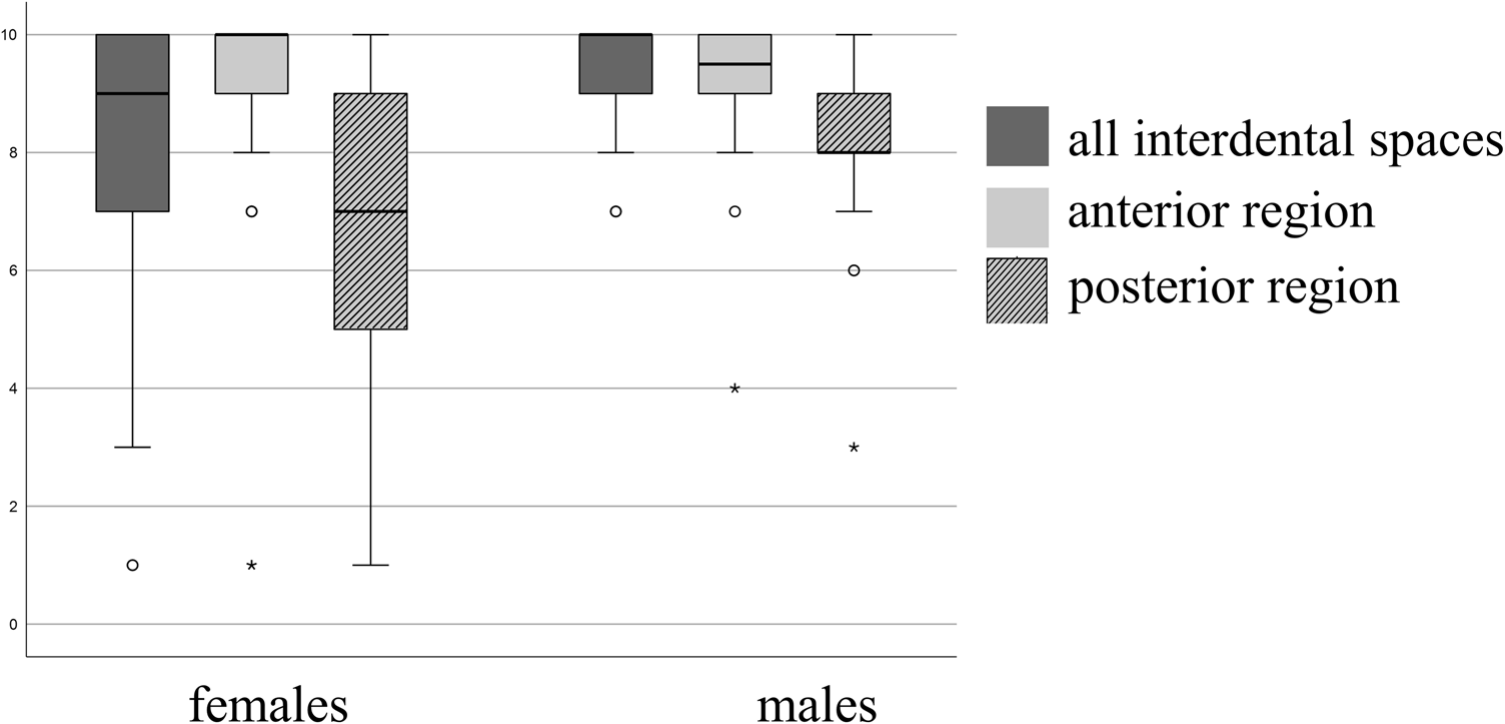
Boxplots of self-assessment of the ability to reach all interdental spaces by gender at T3. The self-assessment is based on an analogue scale from 0: cannot reach interdental spaces, to 10: can reach every interdental space

As shown in Table 2, neither at baseline nor at the end of the study the correct flossing performance was influenced by gender (T1: 1.6% vs. 3.3%; T3: 48.4% vs 36.7%; female vs male, respectively; *p* = n.s), see also Table 2. Despite equal ability to perform the correct flossing technique, however, females rated themselves lower in self-assessment (Fig. 6).

## Discussion

Apart from the already mentioned problem of whether or not to recommend routine flossing, studies dating back to the 1970s already showed that participants were unable to floss properly. One study revealed an average of only 1.5 teeth correctly flossed in 215 participants aged 18–35 years [24], and another showed an average of only one tooth correctly flossed in a group of 240 participants aged 18–65 years [25]. A more recent study confirmed these results, showing that only one in 100 participants was able to clean all interdental spaces using the correct technique [23]. One possible explanation for this might be that flossing is not adequately explained and taught, leading many individuals to lack the manual dexterity required to integrate effective flossing into their daily oral hygiene routine.

This is supported by the results of our questionnaires, as only 24 participants stated that they had received instruction on correct flossing and many considered flossing to be difficult, time-consuming and the molars difficult to reach. In addition, approximately half of the participants reported flossing less than once a week, further illustrating the gap between recommended dental hygiene practices and actual behaviours.

Accordingly, this lack is also mirrored in the baseline observation data, which shows that the participants were only able to floss correctly to a very limited extent. A promising result was that at least almost half of the participants were able to reach all the interdental spaces before receiving the video instruction. However, in view of the technique used, the results were only modest, as the approach was not necessarily systematic and only two participants mastered a correct flossing technique in the entire dentition.

The remaining participants struggled to reach all interdental spaces, with the ability to floss decreasing significantly from the anterior teeth to the molars, with an inconsistent and unsystematic approach resulting in some interdental spaces being flossed several times, while others were not flossed at all. In addition, horizontal sawing movements (41%) and in–out movements (32%) were predominantly used, with only 14% of interdental spaces being correctly flossed vertically.

The relevance of a correct flossing technique for plaque removal and how much plaque needs to be removed to achieve a clinical benefit has not been adequately investigated yet. However, it can be assumed, that with the "in–out" technique or "horizontal sawing" movement plaque cannot be removed effectively.

The video instruction led to significant improvements in flossing, which were reflected in all parameters of the participants. In particular, increased self-confidence in flossing was noted. Participants reported easier handling and better access to posterior interdental spaces, but also estimated it to be much more time-consuming.

The increase in self-confidence was also reflected in the actual improvement of flossing skills. The video instruction taught a systematic approach that resulted in more interdental spaces being reached, especially in the posterior region. In addition, participants learned to implement the correct technique, regardless of region. At the end of the study, 42 participants were able to floss correctly throughout their entire dentition.

The studies from the 1970s achieved similarly good results with video instructions and also showed, that even laypersons are able to use floss correctly with appropriate instruction. The aim of the first study [24] was to investigate the effects of individual in comparison to group instructions. During the group instruction, participants received a detailed 45 min televised instruction in which they performed the flossing procedures on their own teeth in unison with the televised instruction. The individual instruction group received individual chair-side instructions that covered the same content and lasted the same amount of time. Participants in yet another group received no instruction. As expected, the participants in the control group without instruction did not improve and were only able to floss 12% of the teeth correctly, while participants in the chair-side instruction and televised instruction were able to floss 66 and 77% of the teeth correctly, which corresponded to an improvement of 83 and 84%, respectively. Neither method had a significant advantage over the other; thus, the authors concluded that group instruction is a resource-saving alternative to individual instruction.

The follow-up study [25] examined whether the combination of televised and chair-side instruction could have an additional benefit. The same television film was used and the participants were asked again to self-perform flossing while watching the television film. After one week, the same chair-side instruction was given as in the previous study and the participants self-performed flossing during the instruction session. Similar to the first study, the first instruction led to 74% of the teeth being flossed correctly, while the second instruction improved the result to 94%. However, as the authors discuss, it remains unclear whether the different instruction methods, the repeated instruction, the repeated instruction with practice time or even the further practice time alone are the reason for the improvement.

Even though the authors of [24; 25] emphasise the efforts saved by group instruction, the time required for the 45-min film was much longer than in the present study. It is therefore an encouraging finding that a significant improvement in flossing skills can be achieved even with much shorter instruction times. The present study also showed that instruction at the same practice time intervals of one week each as in [25] leads to an improvement in performance in the first interval, but that a subsequent practice interval can lead to additional improvement, even if no further instruction is given.

However, neither study [24, 25] examined whether self-application of floss in the sense of hands-on instruction has an advantage over visual instruction by means of a film. Contrary to our expectations, however, this was not the case in the present study. Although the participants practiced with the instructor after the video instruction until the flossing technique was correct, this did not lead to any additional benefit. The reasons for this cannot be readily deduced from our study. Perhaps the students were intellectually capable of transferring what they had seen in the video into an adequate motor response, so that the one-time practice did not result in an additional performance gain. It could also be due to the lack of practice time and the fact that the participants' ability to absorb new information had already been exhausted because the additional practical training took place immediately after the video instructions. It is also possible that those who were interested in the study were also particularly motivated to learn how to use dental floss, so that implementing instructions was not that difficult. It would therefore be interesting to investigate whether hands-on training might be helpful for other groups of individuals, such as older age groups.

Among other factors, the frequency of use at home prior to the study could have had an effect on learning. Contrary to our expectations, however, the number of those who were able to flow correctly was higher among the inexperienced users. This can perhaps be explained by the fact that new learning could be more unbiased, while those who had already used floss more frequently at home not only had to learn new things but also had to reorganise old and possibly ingrained movement patterns.

On the other hand, and this was very surprising, prior instruction had no effect on the ability to apply the correct flossing technique. Those, who reported receiving prior instruction reached more interdental spaces from the beginning than those who did not. However, when it came to implementing the correct technique, 33 people without prior instruction and 11 people with prior instruction (not significant) were able to implement the correct technique. This shows that improvement of interdental hygiene is possible with good video instruction, regardless of prior instruction.

Another finding was that both males and females performed similarly in using the correct flossing technique. However, females rated themselves lower than males in the self-report. This suggests that it might be of interest to consider gender-specific teaching strategies.

Despite all improvements, a group of people remained, who had only partially learned how to floss. The question of whether their motivation, manual dexterity or visual receptivity are explanatory factors cannot be answered within this study. It is possible that other instruction methods need to be developed for this group. Another reason could have been a lack of interest in learning the technique, as these participants may have only taken part in the study because of the expense allowance.

Some limitations of the present study need also to be discussed. First of all, the extent to which the present results can be generalised. In the present study a group of students took part whose level of education implied a rather high capacity of comprehension. Thus it is unclear to which extend the given results can be transferred to other groups of people. Secondly, the criteria for the correct flossing performance were set comparably high, therefore only two participants were able to fully perform the correct technique at the beginning and not even half of them later, which represents a relatively low success rate. However, we considered the evaluation of individual parameters as partial assessments to be meaningless because clinically relevant results can only be achieved if the criteria are implemented in the entire dentition. Since there was no plaque or gingival index collected in this study, no conclusion can be drawn about the effectiveness of correctly learned flossing. Still the present study can be considered as a basis for further investigations into whether such improved flossing care actually leads to the prevention of plaque-associated diseases.

## Conclusions

Summing up, the results show that even a short video instruction can lead to a significant improvement in flossing skills but that additional hands-on instruction does not significantly improve the results. Thus, the instruction video is a potentially effective and easy-to-use tool for improving interdental hygiene both for individual prophylaxis in the dental practice and for group prophylaxis.

## Supplementary information

Below is the link to the electronic supplementary material.

Supplementary file1 (DOCX 16 KB)

## Data Availability

No datasets were generated or analysed during the current study.
