# Supplementary material for: Impact of video instructions and additional hands-on instructions on the dental flossing performance – an observational study
Source: Clin Oral Investig. 2024 Nov 28;28(12):666. doi: 10.1007/s00784-024-06070-x (PMC11604823; doi:10.1007/s00784-024-06070-x)
Supplement: Supplementary file 1 — Supplementary file1 (DOCX 16 KB) [file 784_2024_6070_MOESM1_ESM.docx]

**Supplementary Table 1**: Compilation of all parameters used (number (n) or median (95%CI)) according to type of instruction and statistical comparison of the two groups (interval-scaled data: Mann-Whitney-U-test; nominal-scaled data: chi-squared test)

|  | Video instruction+ hands on instruction | Video instruction | p value |
| --- | --- | --- | --- |
| Number of participants | 47 | 47 |  |
| Age (yr) | 24 (22;25) | 24 (23;24) | p = 0.443 |
| Gender (n) | female: 30 | female: 34 | p = 0.507 |
|  | male: 17 | male: 13 |  |
| Questionnaires | | | |
| Flossing frequency prior to the study (n) | ≥ once a week: 22  < once a week: 25 | ≥ once a week: 21  < once a week: 26 | p = 1.000 |
| Prior flossing instructions (n) | yes: 11 / no: 36 | yes: 13 / no: 34 | p = 0.813 |
| Floss is easy to use (n) | T1: 6 (5;7) | T1: 6.5 (5;7) | p = 0.686 |
|  | T2: 8 (7;8) | T2: 7 (7;8) | p = 0.559 |
|  | T3: 9 (8;9) | T3: 8 (8;9) | p = 0.447 |
| Flossing is time consuming (n) | T1: 5 (3;7) | T1: 5 (4;7) | p = 0.761 |
|  | T2: 6 (4;7) | T2: 6.5 (5;8) | p = 0.629 |
|  | T3: 6 (4;7) | T3: 5.5 (4.5;7) | p = 0.994 |
| I reach all interdental spaces (n) | T1: 6 (4;8) | T1: 6.5 (4;9) | p = 0.507 |
|  | T2: 9 (9;9) | T2: 8.5 (7;9) | p = 0.276 |
|  | T3: 9 (9;10) | T3: 9 (8;10) | p = 0.342 |
| I reach all anterior interdental spaces (n) | T1: 9 (8;10) | T1: 9 (7;9)) | p = 0.131 |
|  | T2: 9 (8;10) | T2: 9 (9;10) | p = 0.787 |
|  | T3: 10 (9;10) | T3: 9 (9;10) | p = 0.337 |
| I reach all posterior interdental spaces (n) | T1: 4 (3;5) | T1: 5 (4;6.5) | p = 0.438 |
|  | T2: 7 (6;7) | T2: 7 (5;7.5) | p = 0.942 |
|  | T3: 8 (7;8) | T3: 8 (7;8) | p = 0.628 |
| Observational data | | | |
| Reached interdental spaces - all (%) | T1: 92.3 (69.2;96.2) | T1: 92.3 (76.9;96.2) | p = 0.991 |
|  | T2: 96.2 (92.3;100) | T2: 96.0 (92.3;96.2) | p = 0.836 |
|  | T3: 96.2 (92.3;100) | T3: 96.2 (92.3;96.2) | p = 0.749 |
| Reached interdental spaces - anterior (%) | T1: 100 (90.0;100) | T1: 100 (90.0;100) | p = 0.702 |
|  | T2: 100 (100;100) | T2: 100 (100;100 | p = 0.510 |
|  | T3: 100 (100;100) | T3: 100 (100;100 | p = 0.953 |
| Reached interdental spaces - premolars (%) | T1: 100 (87.5;100) | T1: 100 (87.5;100) | p = 0.648 |
|  | T2: 100 (100;100) | T2: 87.5 (87.5;100) | p = 0.129 |
|  | T3: 100 (100;100) | T3: 87.5 (87.5;100) | p = 0.234 |
| Reached interdental spaces - molars (%) | T1: 87.5 (37.5;100) | T1: 87.5 (62.5;100) | p = 0.839 |
|  | T2: 100 (87.5;100) | T2: 100 (87.5;100) | p = 0.516 |
|  | T3: 100 (87.5;100) | T3: 100 (87.5;100) | p = 0.517 |
| Systematic approach score | T1: 17 (12;22) | T1: 21 (17;23) | p = 0.312 |
|  | T2: 22 (21;24) | T2: 23 (22;24) | p = 0.831 |
|  | T3: 23 (22;24) | T3: 23 (23;24) | p = 0.772 |
| Vertical technique (%) | T1: 0 (0;0) | T1: 0 (0;0) | p = 0.668 |
|  | T2: 84.6 (0;100) | T2: 100 (33.3;100) | p = 0.251 |
|  | T3: 96.2 (84.6;100) | T3: 100 (92.3;100) | p = 0.344 |
| Time (s) | T1: 92 (77;110) | T1: 101 (87;114) | p = 0.821 |
|  | T2: 219 (195;291) | T2: 214 (190;243) | p = 0.558 |
|  | T3: 230 (205;251) | T3: 210 (197;263) | p = 0.765 |
| Accumulation of criteria for the correct use of dental floss | | | |
| 24 or more interdental spaces reached (n) | T1: yes: 26 / no: 21 | T1: yes: 25 / no: 22 | p = 1.000 |
|  | T2: yes: 30 / no: 17 | T2: yes: 30 / no: 17 | p = 1.000 |
|  | T3: yes: 32 / no: 15 | T3: yes: 33 / no: 14 | p = 1.000 |
| + mesial/distal adaption (n) | T1: yes: 6 / no: 41 | T1: yes: 4 / no: 43 | p = 0.740 |
|  | T2: yes: 27 / no: 20 | T2: yes: 26 / no: 21 | p = 1.000 |
|  | T3: yes: 31/ no: 16 | T3: yes: 31 / no: 16 | p = 1.000 |
| + vertical technique in more than 90 % of the interdental spaces reached (n) | T1: yes: 1 / no: 46 | T1: yes: 2 / no: 45 | p = 1.000 |
|  | T2: yes: 14 / no: 33 | T2: yes: 16 / no: 31 | p = 0.825 |
|  | T3: yes: 21 / no: 26 | T3: yes: 22 / no: 25 | p = 1.000 |
| + correct handling (n) | T1: yes: 0 / no: 47 | T1: yes: 2 / no: 45 | p = 0.495 |
|  | T2: yes: 14 / no: 33 | T2: yes: 16 / no: 31 | p = 0.825 |
|  | T3: yes: 20 / no: 27 | T3: yes: 22 / no: 25 | p = 0.836 |
